# Mean Platelet Volume as a Potential Marker of Large Vessel Occlusion and Predictor of Outcome in Acute Ischemic Stroke Patients Treated with Reperfusion Therapy

**DOI:** 10.3390/life11060469

**Published:** 2021-05-24

**Authors:** Aleksander Dębiec, Aleksandra Pogoda-Wesołowska, Piotr Piasecki, Adam Stępień, Jacek Staszewski

**Affiliations:** 1 Clinic of Neurology, Military Institute of Medicine, Szaserow 128, 04-141 Warsaw, Poland; apogoda@wim.mil.pl (A.P.-W.); astepien@wim.mil.pl (A.S.); jstaszewski@wim.mil.pl (J.S.); 2 Department of Interventional Radiology, Military Institute of Medicine, Szaserow 128, 04-141 Warsaw, Poland; ppiasecki@wim.mil.pl

**Keywords:** stroke, mean platelet volume (MPV), mechanical thrombectomy, thrombolysis, large vessel occlusion

## Abstract

(1) Background: An early diagnosis of a large vessel occlusion (LVO) is crucial in the management of the acute ischemic stroke (AIS). The laboratory predictors of LVO and a stroke outcome remain unknown. We have hypothesized that high MPV—a surrogate marker of the activated platelet—may be associated with LVO, and it may predict a worse AIS outcome. (2) Methods: This was a retrospective study of 361 patients with AIS who were treated with thrombolysis (tPA, 65.7%) and/or mechanical thrombectomy (MT, 34.3%) in a tertiary Stroke Center between 2011 and 2019. (3) Results: The mean MPV in the cohort was 9.86 ± 1.5 fL (1st–4th quartiles: <8.8, >10.80 fL). Patients in the 4th quartile compared to the 1st had a significantly (*p* < 0.01) more often incidence of an LVO related stroke (75% vs. 39%) and a severe stroke manifestation with a higher RACE score (5.2 ± 2.8 vs. 3.3 ± 2.4), NIHSS at baseline (mean ± SD, 14 ± 6.5 vs. 10.9 ± 5.2), and NIHSS at discharge (6.9 ± 7 vs. 3.9 ± 3.6). A multivariate analysis revealed that quartiles of MPV (OR 1.4; 95%CI 1.2–1.8) significantly predicted an LVO stroke, also after the adjustment for RACE < 5 (OR 1.4; 95%CI 1.08–1.89), but MPV quartiles did not predict a favorable stroke outcome (mRS ≤ 2) (OR 0.89; 95%CI 0.7–1.13). (4) Conclusion: Our data suggest that MPV is an independent predictor of LVO in patients with an acute ischemic stroke.

## 1. Background

The treatment time remains one of the most important factors in the interventional ischemic stroke (IS) treatment [1]. An earlier endovascular or recombinant tissue plasminogen activator therapy (tPA) is related to the substantially better IS outcomes [2]. Every 30 min of delay decreases the chance of a good outcome by 10%. One of the most important pre-hospital stroke types of management is to diagnose a large vessel occlusion (LVO) patient who could benefit from the mechanical thrombectomy (MT) treatment properly and should be directly transported to the nearest thrombectomy–ready hospital. Strokes due to LVO are responsible for more than one-third of those severely presenting the acute cerebral ischemia events, three-fifths of the poststroke dependence and death, and more than nine-tenths of the poststroke mortality [3]. 

The interfacility transfers may cause significant delays and result in the worse outcomes compared to the direct transport to the endovascular facility. Some clinical stroke severity scales, e.g., The National Institutes of Health Stroke Scale (NIHSS); Rapid Arterial Occlusion Evaluation Scale (RACE); or The Vision, Aphasia, and Neglect (VAN) are strongly associated with the presence of LVO, but they are time consuming and require some experience in their assessment [4]. Although RACE ≥ 5 has 85% of sensitivity and 68% specificity, it can correctly classify 71% of LVO patients; therefore, more easily applied, less time consuming, and more accurate measures would be valuable to diagnose the LVO patients at the pre-hospital level correctly [5]. In addition, the risk of a misdiagnosis is much greater when presenting neurologic complaints are mild or nonspecific. Unfortunately, the blood biomarkers which could serve as feasible and early-stage predictors of LVO have not been identified so far because the mechanisms underlying the acute LVO are still largely unknown. 

Hyperactive platelets play a pivotal role in the thrombus formation and propagation, leading to the acute thrombotic events. The mean platelet volume (MPV) is a machine-calculated index of the average platelet size. It correlates with the platelet activation, and its precise measurement may provide clues to both underlying vessel pathology and the most effective interventions aimed at a stroke treatment [6]. We have previously found that the elevated MPV may predict the disabling of the fatal IS in the patients treated with tPA and that a prognostic value of MPV was independent of other well-defined individual risk factors [7]. However, the studies evaluating the association between the MPV and the clinical outcome in the patients following MT have resulted in contradictory results so far. Peng F. et al. reported that an elevated MPV level (higher than the mean 10.4 fL) was an independent predictor of the unfavorable outcome in the LVO anterior circulation stroke treated with MT, but this finding has been not confirmed by other investigators [8,9]. Additionally, the value of MPV in predicting the risk of the secondary hemorrhage in the patients receiving the reperfusion therapy remains unclear [10]. On the basis of the reported higher levels of MPV in the patients with more severe strokes on admission and the lack of the early improvement in these patients following tPA treatment, we have hypothesized that an elevated MPV level on admission may be associated with the acute LVO. 

## 2. Aim

The primary objective of the study was to investigate whether there is an association between the admission of MPV and LVO in the patients with IS treated by means of the reperfusion therapy. The secondary aim was to evaluate the relationship between the admission of MPV and the functional outcome at discharge. 

## 3. Materials and Methods

This retrospective study included 361 patients who were consecutively admitted to a tertiary stroke care center over 9 years (1 January 2011–31 December 2019) due to the anterior circulation IS and treated with the reperfusion therapy: MT according to the AHA/ASA guidelines within 6 h of the symptoms’ onset and/or standard-dose of IV tPA according to the National Institute of Neurological Disorders and Stroke criteria and schedule. However, no upper age limit was applied, and the treatment was allowed up to 4.5 h after the onset, provided that there were no contraindications in either situation [11]. 

We excluded the patients with diseases that could have clinically relevant influence on MPV and raise the risk of tPA complications. These include sepsis, immunosuppression, autoimmune disorders, aplastic anemia, thrombocytopenia, acute leukemia, and drug-induced hypoplasia of marrow. All the patients received a standardized stroke diagnosis according to the national and international guidelines which consisted of clinical examinations, cerebral imaging (computed tomography (CT) scans), and CT arteriography (CTA) of intracranial and extracranial arteries. In all the patients, CT and CTA scans were performed immediately on admission, and control CT was performed 24 h after the admission or earlier in the case of the neurologic deterioration to rule out intracranial bleeding. LVO was recognized according to baseline CTA scans. In the patients treated with MT-modified thrombolysis in cerebral infarction, the (mTICI) grade was calculated from the last angiographic control images, and the mTICI scores of 2b to 3 were considered as a successful revascularization. 

The blood samples for MPV and other biochemical markers were drawn from the antecubital vein at baseline within a mean of 14 ± 7 min of sampling after the admission to the Emergency Department (a mean of 3.25 + 1.2 h after a stroke onset) and the results were available after approximately 30 min; the samples were transported at room temperature and assessed by a Sysmex XN-1000 hematology autoanalyzer (Sysmex Corporation, Kobe, Japan). The samples for MPV were collected in EDTA, and MPV was measured in units of fL (normal range, 7.5–11.5 fL). 

The severity of a stroke was assessed by a trained neurologist immediately on admission to the Emergency Department in NIHSS and RACE scales, and solely in NIHSS within 24 h after MT or tPA administration, and at discharge. To measure a global disability and functional dependence on admission and discharge, we used the modified Rankin scale (mRS) which ranges from 0 (no symptoms) to 6 (death). The efficacy outcome was defined as the rate of a good outcome (mRS ≤ 2) at discharge.

The time of the symptoms onset, the age, the sex, diabetes mellitus, hypertension, dyslipidemia, current smoking, atrial fibrillation (AF), the history of antithrombotic medication, coronary heart disease (CHD), body mass index, a previous stroke or a transient ischemic attack (TIA), BP, and serum glucose were routinely collected on admission. Diabetes mellitus was defined as a non-fasting glucose concentration ≥ 11.1 mmol/L, a fasting glucose level ≥ 7.0 mmol/L, the use of glucose-lowering drugs, or a self-reported history of diabetes. The hypertension was defined as the systolic blood pressure (SBP) ≥ 140 mmHg or the diastolic blood pressure (DBP) ≥ 90 mmHg, any use of antihypertensive drugs, or any self-reported history of hypertension. Dyslipidemia was defined as a serum low-density lipoprotein (LDL) cholesterol ≥ 3.6 mmol/L, high-density lipoprotein (HDL) cholesterol ≤ 1.0 mmol/L, triglyceride (TG) level ≥ 1.7 mmol/L, the use of lipid-lowering drugs, or a self-reported history of dyslipidemia. The carotid artery stenosis was recognized if either the occlusive or stenotic (≥50% diameter reduction) vascular disease judged to be due to atherosclerosis was found in the clinically relevant or irrelevant extracranial arteries. The Causative Classification of Stroke (CCS) method was used to classify the stroke etiology in accordance with the Stop Stroke Study Trial of Org 10172 in Acute Stroke Treatment criteria [12]. These criteria integrate the results of brain imaging, clinical evaluations, heart/vascular examinations, and work-up for rare causes of stroke. Strokes were categorized into one of the four following categories: large-artery atherosclerosis, cardioembolism, small-vessel occlusion (lacunar stroke), and others (cryptogenic or determined etiologies). If some multiple potential causes existed, then the patient was assigned to the undetermined cause group. The baseline CT brain scans of the IS patients were analyzed for early ischemic changes with The Alberta Stroke Program Early CT Score (ASPECTS) [13]. If there was the evidence of the early signs of brain ischemia in each of ten areas (e.g., loss of gray-white matter differentiation, reduced attenuation or hypodensity, focal swelling, obscuration of the lentiform nucleus, or insular ribbon sign), a point was deducted from the baseline score of ten, ignoring old infarcts. A scan with no ischemia in the MCA territory was scored 10, and a scan with the diffuse involvement of all MCA territories was scored 0.

We have conducted this study in accordance with the Declaration of Helsinki. The electronic database was decoded, and the patient identification data was scrambled to ensure confidentiality; the informed consent was thus exempted. The evaluation of all the imaging studies was blinded from the clinical data. This study was evaluated and approved by the Institutional Review Board of Military Institute of Medicine in Warsaw. 

### Statistical Analysis

All the data were first analyzed for normality of distribution using the Kolmogorov–Smirnov test of normality. The results are shown as the mean ± SD or as counts and percentages. The baseline characteristics of the individuals were summarized according to the quartiles’ distribution of MPV. The first group contained the lowest 25% of MPV values and served as a reference; the second contained the next lowest 25% of numbers (up to median); the third contained the second highest 25% of number (above the median); and the fourth included the highest 25% of values. The clinical characteristics of the four groups were compared using one-way ANOVA for continuous variables; the chi-squared test was used to compare the categorical parameters. The comparisons between normally distributed continuous variables were made using ANOVA with a post-hoc analysis of the least significant difference, and the Kruskal–Wallis test with Bonferoni’s post hoc adjustment was used for nonparametric distributed variables. The logistic regression was used to calculate the relation between LVO, favorable stroke outcome, and with quartiles of MPV and other clinical variables. All the variables with *p*-values < 0.05 in univariate analysis were included in the multivariate analysis. To evaluate the MPV and other variables’ power to predict LVO, we also used the receiver operating characteristic (ROC) curves, which compare sensitivity versus specificity across a range of values for the ability to predict a dichotomous outcome. The best cut-off for maximizing sensitivity and specificity according to the Youden index criterion was established [14]. A statistical significance was set at *p*-value < 0.05 for all the analyses. The statistical analyses were performed with the PQStat software (v1.8.0, Poland). 

## 4. Results

### 4.1. Baseline Clinical Characteristics of Studied Cohort

The studied cohort consisted of 361 patients (mean age 70.3 ± 12.5, 51.2% females) of whom 124 patients (34.3%) were treated with MT (32 patients (25%) also received bridge tPA), and 237 (65.7%) underwent IV thrombolysis alone. The most common etiology was a large-artery atherosclerosis (45%); 37% patients were diagnosed with cardioembolism; in the remaining subjects, the stroke was associated with small-vessel disease, or the etiology could not be determined. The majority of patients were functionally dependent (mRS > 2) on admission (n = 230; 64%), and had a severe neurologic deficit (mean ± SD; NIHSS score 13.3 ± 6). Typically, the patients received a reperfusion therapy (onset-to-treatment (OTT): MT and/or tPA) later than 3 h of stroke onset (52.6%; 3.2 ± 1.2 h, interquartile range 1 h 54 min). There was no significant difference (*p* > 0.1) between the patients who received MT or solely tPA with regard to the average age (respectively; 69 ± 12.2 vs. 71 ± 12.6 years), the distribution of sex (52.4% vs. 50.6% females), the frequency of the main vascular risk factors (hypertension 75% vs. 73%, diabetes 25% vs. 21%, AF 42% vs. 35%, CHD 25% vs. 24%, dyslipidemia 32% vs. 28%, smoking 26% vs. 21%, obesity 21% vs. 30%, past stroke 13% vs. 14%) and the frequency of large artery atherosclerotic and cardioembolic strokes (respectively, 51% and 42% vs. 46% and 35%). However, the patients who received MT had higher baseline NIHSS (mean 16.2 ± 5.6 vs. 11.8 ± 5.8; *p* < 0.01) and were more often dependent (84.4% vs. 53.6%, *p* < 0.01). This group also had a longer mean OTT (4 ± 1.3 vs. 3 ± 1 h, *p* < 0.05) and a higher mean MPV (10.5 ± 1.3 vs. 9.5 ± 1.4; *p* < 0.01).

### 4.2. Characteristics of Studied Subjects Stratified into MPV Quartiles

**Figure 1:**
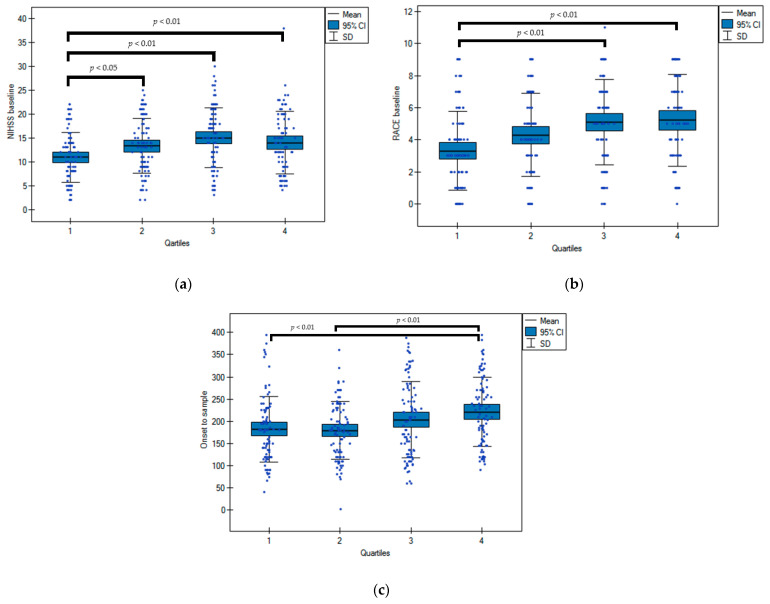
Baseline NIHSS (**a**), RACE (**b**) scores, and delay from stroke onset to blood sample (**c**) by MPV quartiles. One-way analysis of variance (ANOVA) by ranks.

The mean MPV in the cohort was 9.86 ± 1.5 fL. The median MPV was 9.9 fL (intraquartile range 8.8–10.8), and the subjects were divided according to MPV distribution into: the first quartile ≤ 8.8 fL, the second 8.8 to ≤9.9 fL, the third 9.9 to ≤10.8 fL, highest > 10.80 fL. There was no difference between mean MPV values between gender (females 9.81 ± 1.39 vs. males 9.92 ± 1.61 fL, two-way ANOVA test *p* = 0.48). Additionally, the mean age, sex distribution, and frequency of atherothrombosis risk factors were similar across quartiles. The patients in the highest quartile compared to the lowest had a more severe stroke manifestation with a higher NIHSS at baseline, after 24 h, and at discharge, and they also scored a higher RACE at baseline (5.2 ± 2.8 vs. 3.3 ± 2.4, *p* < 0.01). They were also more frequently diagnosed with LVO and had a longer delay from the stroke onset to blood sample (Table 1, Figure 1). There was no difference between quartiles with regard to the frequency of antiplatelet, anticoagulant, or hypolipidemic agents used before the index stroke. In the univariate analysis, baseline NIHSS (OR 0.8; 95%CI 0.76–0.84), age (OR 0.97; 95%CI 0.95–0.98), MPV quartile (OR 0.71; 95%CI 0.58–0.86), OTT (OR 0.99; 95%CI 0.994–0.999), LVO (OR 0.46; 95%CI 0.3–0.7) were all related to a favorable stroke outcome (mRS ≤ 2) at discharge (*p* < 0.01). However, the baseline NIHSS (OR 0.8; 95%CI 0.74–0.86), age (OR 0.97; 95%CI 0.95–0.99), and OTT (OR 0.99; 95%CI 0.992–0.999) remained the only significant predictors of an outcome in the multivariate analysis, while MPV quartiles and LVO lost their statistical significance (respectively; OR 0.89; 95%CI 0.7–1.13; OR 1.16; 95%CI 0.64–2.11). 

Among all the analyzed clinical factors, only the baseline NIHSS, RACE, MPV, quartiles of MPV and CHD were related to the risk of LVO in univariate analysis (Table 2). 

**Figure 2:**
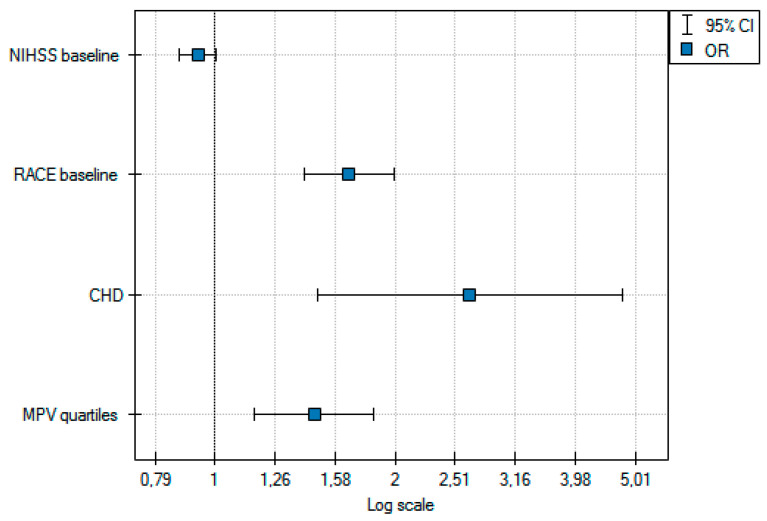
Multivariate analysis of factors related to large vessel occlusion (LVO).

A multivariate analysis revealed that quartiles of MPV (OR 1.4; 95%CI 1.2–1.8), RACE (OR 1.6; 95%CI 1.4–1.9), and CHD (OR 2.6; 95%CI 1.4–4.7) significantly predicted a stroke due to LVO (Figure 2). These results did not change significantly after the adjustment for a delay from the stroke onset to the blood sample. 

Interestingly, also in the patients with less severe symptoms of stroke scoring RACE <5 p on admission, after the adjustments for CHD and a delay from the onset to sample, MPV quartiles (OR 1.4; 95%CI 1.08–1.89) still significantly predicted LVO in a multivariate analysis (*p* < 0.01). 

**Figure 3:**
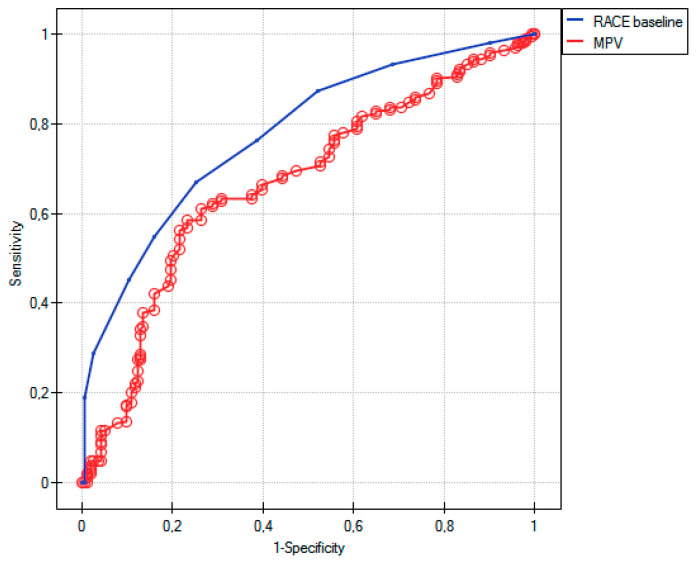
ROC curves for various cut-off levels of RACE and MPV in differentiating between LVO and non-LVO related ischemic strokes.

In the ROC analyses, the RACE score had a better diagnostic accuracy for predicting LVO (AUC 0.77; SE 0.02; −95%CI 0.72; +95%CI 0.82) compared to MPV (AUC 0.66; SE 0.02; −95%CI 0.61; +95%CI 0.72) (*p* < 0.01) (Figure 3). A RACE scale ≥ 5 and MPV ≥ 10.2 fL had the highest sensitivity (respectively, 0.66 vs. 0.61) and specificity (0.74 vs. 0.73), and demonstrated a positive predictive value (PPV) and a negative predictive value (NPV) of 0.75 and 0.65 vs. 0.74 and 0.61 for the diagnosis of LVO.

## 5. Discussion

The stroke is a leading cause of major long-term disability and an enormous source of global disease burden. There is an urgent need to establish prognostic models of clinical, imaging, and laboratory variables that can help clinicians to qualify for MT and predict a stroke outcome at an early stage [15]. Considering the limited availability of comprehensive stroke centers and time sensitivity of the reperfusion therapies in stroke, it is of the utmost importance to discriminate LVO from other ischemic strokes and triage the early LVO patients who are eligible for the reperfusion therapies. Our study revealed that a widely and easily available assessment of MPV can be useful in the diagnosis of LVO in both the prehospital and the in-hospital settings. A high admission MPV was observed more often in the subjects with an LVO stroke, and the quartiles of MPV predicted LVO irrespectively of the severity of a stroke at admission. The prognostic value of MPV was independent of other well-defined individual risk factors. Moreover, a univariate analysis showed that higher MPV levels on admission were associated with a more severe stroke outcome with functional dependence; however, this was not confirmed after the adjustment for other confounders. 

The emergency triage of suspected patients who had a stroke is usually based only on the clinical examination itself, and an efficient and rapid assessment is the most important key factor in a stroke outcome. The multiple stroke scales, e.g., NIHSS, RACE, Cincinnati Prehospital Stroke Severity Scale (CPSS), the Los Angeles Motor Scale (LAMS), have been validated for clinical use and LVO prediction in the stroke field; however, the majority of them are subjective and time-intensive, and a recent meta-analysis reported that the scales have a low predictive value for the presence of LVO (between 35% and 50%) [16]. NIHSS may be predictive for LVO in the anterior circulation, but it is limited by time dependence, the experience of the examining medical staff, and the patient’s cooperation, and still it does not have a widely accepted a cutoff point. [17] Additionally, the LAMS and CPSS do not include all the cortical signs, while the RACE scoring system requires six NIHSS items to score, thereby making it complex and time-consuming [18]. Therefore, apart from the predictive value, the resources and the cost of training required to introduce and keep the efficiency of such instruments in prehospital stroke care are crucial. The RACE scale has a high predictive value for LVO with the established AUC of 0.82 in the original report [19]. In our analysis, RACE turned out to be a more reliable marker (AUC 0.77) compared to MPV (AUC 0.66). Similarly to our data which revealed that RACE ≥ 5 had 66% sensitivity and 74% specificity, different studies demonstrated that RACE ≥ 5 had sensitivity between 55–85% and specificity 68–90% [20]. We showed that MPV ≥ 10.2 fL had an acceptable sensitivity (61%) and specificity (73%) for the diagnosis of LVO. Importantly, the quartiles of MPV were strongly related to the risk of LVO—also, after the adjustment for a delay from the stroke onset to blood sampling, in the patients with clinically minor strokes, and both in cardioembolic and atherosclerotic strokes. If confirmed in further studies, MPV could be incorporated in clinical assessment scales to help increase the predictive value for LVO, e.g., in centers without CTA or even at a prehospital scene. 

In our previous study, we showed that MPV could be an early and easily measured prognostic factor in the AIS patients treated with tPA [7]. In the present study which involved the patients also treated with MT, those in the highest MPV quartile compared to the lowest also had more severe stroke symptoms evaluated in NIHSS at baseline and discharge, but MPV was neither predictive for mortality nor functional stroke outcome in the multivariate analysis. Similar data have been recently published by Sabenca et al. who did not find the association between the baseline MPV value and the clinical outcome within 90 days in the anterior circulation stroke and LVO patients submitted to MT [9].

Furthermore, the measurement of MPV has a prognostic value in stroke prediction among patients with a history of cerebrovascular disease. According to the PROGRESS (Perindopril Protection Against Recurrent Stroke Study), elevated MPV was an independent predictor of stroke in subjects after TIA or stroke with a 12% higher risk with each 1-fL increase in usual MPV [21]. 

Sengeze et al. studied the relationship between MPV and the success of endovascular recanalization in the acute middle cerebral artery (MCA) and demonstrated that there was not a statistical correlation between the first-pass thrombectomy success of MCA M1 occlusions and the MPV values [22]. Additionally, the value of MPV in predicting the risk of a secondary hemorrhage in the patients receiving the reperfusion therapy still remains unclear [10]. Taking into account the number of well-established predictors of the clinical outcome and mortality following MT (e.g., age, revascularization status, parenchymal hemorrhage, NIHSS score on admission, previous stroke, delay from onset to reperfusion, experience of the operator), it is unlikely that a single marker such as baseline MPV could independently predict a stroke outcome [23].

The platelet morphology and function play vital roles in the pathogenesis of many diseases related to thrombosis, endothelial dysfunction, coagulation, and inflammation. However, the pathophysiological mechanism whereby MPV is associated to the prognosis and distribution of IS is not yet clear. Large platelets are enzymatically and metabolically more active than smaller ones; they secrete and express more mediators: adhesive proteins (fibrinogen, thrombospondin), growth factors (platelet-derived growth factor, transforming growth factor-β), along with the chemotactic and mitogenic factors (platelet factor 4, coagulation factor V, and factor XI), and cytokine-like factors (interleukin-1 and CD40 ligand) [24]. It may result in platelets adhesion, aggregation, and a pro-atherosclerotic effect with the increased risk of thrombus formation after the atherosclerotic plaque rupture and thromboembolic risk in the patients with atrial fibrillation [25,26,27]. These findings support the notion that enhanced MPV might reflect a greater prothrombotic state, which could result in more severe atherthrombotic or cardioembolic stroke. Furthermore, the increased MPV values are associated with shortened bleeding time; however, whether it may influence treatment response, e.g., to antiplatelet agents, is not clear, and it requires further analysis [28]. Elevated MPV and its prognostic value of cardiovascular risk is closely associated with increased sympathetic activity [29]. A stroke causes changes in the autonomic nervous system that leads to mentioned increased sympathetic and reduced parasympathetic activity, but the exact impact of that phenomenon on the course of IS is not well known [30]. 

Several limitations of this study should be noticed. Our analyses include only patients whom were confirmed to have the ischemic stroke. Patients suspected of having a stroke were not included. Therefore, we were unable to investigate the exact mechanism of MPV in the course of a stroke, and its etiology and location. Since ischemic strokes have different pathophysiologic mechanisms, MPV may have a distinct prognostic value according to each stroke etiology. Another limitation was the lack of a control group (e.g., with hemorrhagic stroke or stroke mimics), and its retrospective and single-center design may have led to bias; thus, our findings should be considered as a generating hypothesis at its best. In addition, our analyses were limited to the short-term in-hospital outcomes, while the prognostic stroke studies used the mRS as the primary outcome measure after three months. Thus, it is unclear how our findings might impact the treatment. We do not know exactly if MPV has a direct contribution to LVO; therefore, further prospective studies in both the acute and the subacute stages of stroke are needed to explain the underlying mechanism. An additional important limitation is that the different factors (such as delay in time from sampling to analysis and the anticoagulant used in the collection tube) can influence normal ranges for the MPV. However, this delay was very short in the study cohort, and all the measurements were performed within 30 min after collection, using the same device. Moreover, in the study, the impact of MPV and platelet activation on the other blood clotting measures, e.g., shortened bleeding time, was not analyzed.

MPV can provide some information on thrombocyte activation; however, the ultimate evidence will be platelet function tests. Therefore, further research is needed to determine the role of circulating platelets in the prognosis of AIS. It should also be emphasized that different automatic cell counters and platelet counting methods, as well as MPV measurements at different time intervals from the onset of stroke, are important factors underlying different results and interstudy contradictions [31].

The availability of MPV with respect to the time from symptom onset to treatment application is a key practical limitation. However, if the prognostic value of MPV in the LVO is confirmed, automated platelet function analyzers may be introduced in the future.

The strengths of the study include the large sample size and the focus on LVO related strokes. To the best of our knowledge, the association between MPV and LVO in the patients receiving the reperfusion therapy has not been previously reported. In conjunction with the clinical (e.g., NIHSS or RACE) and radiological data (e.g., non contrast CT in centers without access to CTA), the MPV measurements that are already available in routine laboratories may represent one of the easiest measurements. It can play a role as a predictor of LVO and help with decision making and selecting the optimal treatment strategies without adding economic burden. The combination of MPV with stroke scores including the vascular risk factors as well as the clinical and the imaging data could increase the predictive value for LVO and an early stroke outcome. However, the methodological problems involved in obtaining an accurate MPV result must be considered, and the results should be carefully standardized. 

## 6. Conclusions

Our data suggest that MPV is an independent predictor of LVO in the patients with an acute ischemic stroke. Interpreting MPV with clinical scales of stroke severity should help more patients with an acute stroke receive the appropriate reperfusion interventions, such as MT or tPA.

## Figures and Tables

**Table 1:** Clinical and radiological characteristics by the MPV quartiles.

	*Quartile 1*	*Quartile 2*	*Quartile 3*	*Quartile 4*	*p* *	Post Hoc **
*n*	92	88	93	88		
Age n (SD)	68.3 (12.5)	69 (13.9)	71.7 (10.8)	72.2 (12.4)	0.1	
Sex (F) n (%)	44 (23.8)	52 (28.1)	50 (27)	39 (21)	0.2	
AF	22 (24)	34 (38.6)	39 (42)	39 (44)	0.01	
Hypertension	62 (68)	68 (77)	75 (81)	59 (68.6)	0.1	
Diabetes	12 (13)	24 (27)	26 (28)	17 (20)	0.06	
Smoking	27 (30)	14 (16)	25 (27)	14 (16)	0.05	
Obesity	124 (38)	73 (23)	54 (17)	105 (33)	0.2	
CHD	24 (26)	21 (24)	23 (25)	19 (22)	0.9	
Dyslipidemia	31 (34)	23 (26)	32 (34)	20 (23)	0.2	
Past stroke	11 (12)	14 (16)	13 (14)	12 (14)	0.9	
OTG (MT) min	257 (77)	223 (62)	246 (95)	246 (73)	0.6	
OTT (tPA) min	168 (62)	172 (62)	178 (63)	178 (65)	0.9	
baseline RACE ≥ 5	24 (26)	39 (44.3)	56 (60.2)	52 (59)	<0.01	
NIHSS baseline	10.9 (5.2)	13.2 (5.7)	15 (6.2)	14 (6.5)	<0.01	1 vs. 2,3,4
NIHSS 24 h	7.4 (5.7)	10.7 (7.6)	12.5 (8.7)	11.8 (8)	<0.01	1 vs. 3,4
NIHSS discharge	3.9 (3.6)	7 (5.8)	8 (7.8)	6.9 (7)	<0.01	1 vs. 2,3,4
mRSdischarge	2.4 (1.8)	3.5 (1.9)	3.6 (1.8)	3.5 (1.9)	<0.01	1 vs. 2,3,4
mRS ≤ 2 discharge	57 (62)	29 (33)	27 (29)	33 (37.5)	<0.01	
In-hospital mortality	8 (8.7)	14 (15.9)	16 (17.2)	13 (14.7)	0.35	
Hyperdense MCA sign	16 (18.3)	25 (30.5)	23 (28.8)	28 (38.9)	0.04	1 vs. 2,3,4
sICH hemorrhage	10 (11)	13 (15)	21 (23)	16 (18)	0.17	
TICI 2b,3 (MT)	11 (85)	13(76)	27 (69)	44 (80)	0.6	
ASPECTS score	9.2 (0.5)	9.1 (0.8)	8.1 (1.2)	8 (1.4)	0.8	
LVO	36 (39.1)	38 (43.2)	54 (58)	66 (75)	<0.01	
Cardioembolic stroke	24 (27)	36 (40)	41 (46)	39 (43)	<0.05	
Atherothrombotic stroke	33 (37)	40 (44)	42 (47)	48 (53)	<0.05	

Values are mean ± SD for quantitative variables and *n* (%) for qualitative variables. Abbreviations: AF, atrial fibrillation; ASPECTS, Alberta stroke program early CT score; CHD, coronary heart disease; LVO, large vessel occlusion; MCA, middle cerebral artery; mRS, Modified Rankin Scale; MT, mechanical thrombectomy; NIHSS, The National Institutes of Health Stroke Scale; OTG, onset to groin; OTT, onset to treatment; sICH, secondary symptomatic hemorrhage; TICI, The thrombolysis in cerebral infarction scale; tPA, tissue plasminogen activator. * ANOVA or Chi^2^, where applicable, ** *p* < 0.05.

**Table 2:** Univariate analysis of factors associated with large vessel occlusion (LVO).

	Odds Ratio	−95%CI	+95%CI	*p* Value
NIHSS baseline	1.11	1.07	1.16	<0.01
RACE baseline	1.51	1.37	1.67	<0.01
Age	1.0	0.98	1.02	0.5
Age ≥ 65	1.11	0.89	1.39	0.3
MPV	1.43	1.22	1.67	<0.01
MPV quartile	1.66	1.36	2.03	<0.01
Hypertension	1.0	0.62	1.61	0.9
AF	1.33	0.86	2.05	0.2
Diabetes	1.57	0.94	2.62	0.08
Smoking	1.4	0.84	2.33	0.2
CHD	2.21	1.33	3.69	<0.01
Dyslipidemia	1.19	0.75	1.88	0.4

Abbreviations: AF, atrial fibrillation; CHD, coronary heart disease; MPV, mean platelet volume; NIHSS, The National Institutes of Health Stroke Scale; RACE, Rapid Arterial Occlusion Evaluation Scale for Stroke.

## Data Availability

The data presented in this study are available on request from the corresponding author. The data is not publicly available due to a still ongoing analysis.
